# Evolution of H3K27me3-marked chromatin is linked to gene expression evolution and to patterns of gene duplication and diversification

**DOI:** 10.1101/gr.162008.113

**Published:** 2014-07

**Authors:** Robert K. Arthur, Lijia Ma, Matthew Slattery, Rebecca F. Spokony, Alexander Ostapenko, Nicolas Nègre, Kevin P. White

**Affiliations:** 1 Department of Ecology and Evolution, University of Chicago, Chicago, Illinois 60637, USA;; 2 Institute for Genomics and Systems Biology, University of Chicago and Argonne National Laboratory, Chicago, Illinois 60637, USA;; 3 Department of Human Genetics, University of Chicago, Chicago, Illinois 60637, USA;; 4 Department of Biomedical Sciences, University of Minnesota Medical School, Duluth, Minnesota 55455, USA;; 5 Department of Natural Sciences, Baruch College, City University of New York, New York 10010, USA;; 6 Université de Montpellier 2 and INRA, UMR1333 DGIMI, F-34095 Montpellier, France

## Abstract

Histone modifications are critical for the regulation of gene expression, cell type specification, and differentiation. However, evolutionary patterns of key modifications that regulate gene expression in differentiating organisms have not been examined. Here we mapped the genomic locations of the repressive mark histone 3 lysine 27 trimethylation (H3K27me3) in four species of *Drosophila,* and compared these patterns to those in *C. elegans*. We found that patterns of H3K27me3 are highly conserved across species, but conservation is substantially weaker among duplicated genes. We further discovered that retropositions are associated with greater evolutionary changes in H3K27me3 and gene expression than tandem duplications, indicating that local chromatin constraints influence duplicated gene evolution. These changes are also associated with concomitant evolution of gene expression. Our findings reveal the strong conservation of genomic architecture governed by an epigenetic mark across distantly related species and the importance of gene duplication in generating novel H3K27me3 profiles.

While transcriptional regulation has long been recognized as a significant target of evolutionary change (King and Wilson 1975), the specific mechanisms behind regulatory divergence have been difficult to dissect (Carroll 2008). In most cases, research has focused on recognizable *cis*-elements where transcription factors bind in a sequence-specific manner (Borneman et al. 2007; Bradley et al. 2010; Dowell 2010; Ni et al. 2012). Changes to either a transcription factor’s DNA-binding properties or transcription factor binding sites (TFBS) enable the evolution of differential regulation, and numerous examples of this phenomenon have been observed (Ludwig et al. 2005; Shultzaberger et al. 2012).

Whereas changes in *cis*-regulatory elements have been implicated in phenotypic and specifically morphological changes (Carroll 2008; Stern and Orgogozo 2008), other components of transcriptional regulation such as the chromatin environment have been scarcely explored (Lenhard et al. 2012). Histone modifications, which are chemical alterations of the histone spools upon which DNA is threaded, constitute one of the best-described elements of chromatin state (Zhou et al. 2011). These modifications can act directly or indirectly (through recruited enzymes) to alter DNA accessibility, thereby controlling other DNA–protein interactions (Campos and Reinberg 2009). Unlike TFBSs, however, histone modifications are not necessarily easily localizable to particular sequence elements, making their evolution difficult to study (Delest et al. 2012).

Histone 3 lysine 27 trimethylation (H3K27me3) is one of the best-known histone modifications, in terms of both its biogenesis and effects. H3K27me3 is associated with complex *cis*-regulatory elements called Polycomb Response Elements (PREs) (Delest et al. 2012). Unlike TFBSs, PREs are compound regulatory elements composed of multiple, sometimes partially redundant, sequence elements (Delest et al. 2012). Although the exact biochemical binding site composition and structural constraints upon PREs are not completely resolved, a group of coordinately binding proteins called Polycomb group (PcG) factors recruit Polycomb Repressive Complexes, which are necessary to deposit and maintain H3K27me3 and have been associated genome wide with H3K27me3 domains (Lanzuolo and Orlando 2012).

The effect of H3K27me3 is unambiguously and strongly repressive (Filion et al. 2010; Kharchenko et al. 2011; Li et al. 2014). H3K27me3-associated loci have been proposed to congregate in silenced foci called Polycomb factories, which inhibit transcription by preventing access to RNA polymerase II and other *trans*-factors (Bantignies et al. 2011; Sexton et al. 2012). This repressive state is both temporally and spatially dynamic (Akkers et al. 2009; Filion et al. 2010; Kharchenko et al. 2011; Nègre et al. 2011).

Accordingly, the PcG proteins and H3K27me3 have been shown to be necessary for differentiation and maintenance of cell type identity in organisms across eukaryotes (Bernstein et al. 2006; Feng and Jacobsen 2011). Dysregulation of H3K27me3 has also been implicated in the genesis and progression of cancer (Chi et al. 2010; Ellinger et al. 2012). These results indicate that H3K27me3 plays a role in the creation and maintenance of cell type-specific programs of transcriptional control for a wide variety of species and cell fates.

Given its close association with the process of differentiation, the extent to which H3K27me3 domains are conserved across species is of great interest (Shubin et al. 2009; Cain et al. 2011). We sought to examine the rate of evolutionary change in H3K27me3 between four species of the well-characterized *Drosophila* clade, the mechanisms by which such change might occur, and the possible consequences of H3K27me3 evolution for nearby gene expression.

In order to investigate the evolution of H3K27me3 patterns across the genome, we performed chromatin immunoprecipitation followed by sequencing (ChIP-seq) for H3K27me3 in four species, *D. melanogaster*, *D. simulans*, *D. yakuba*, and *D. pseudoobscura*, with divergence times ranging from less than 5 million years (Myr) to more than 35 Myr (Tamura et al. 2004; Obbard et al. 2012). To determine whether and how H3K27me3 changes might alter gene expression, we also performed RNA sequencing (RNA-seq) in all species. For all analyses we used white prepupae for ease of developmental synchronization between species and because there has been extensive previous work characterizing genome-wide regulatory evolution at this developmental stage (Rifkin et al. 2003; Gu et al. 2004; Ni et al. 2012). We find that for single-copy orthologous genes, H3K27me3 signal is strongly conserved in even distantly related species. However, duplicated gene orthologs exhibit much greater divergence in H3K27me3. Moreover, different kinds of duplicates appear to have very different rates of H3K27me3 and expression divergence, indicating functional distinctions in the epigenetic consequences of different gene duplication mechanisms.

## Results

### Evolutionary stasis of H3K27me3 levels across single-copy genes in *Drosophila*

We examined H3K27me3 signal at the white prepupal stage of development, a tightly defined, 20-min window at the beginning of pupariation. Two biological replicates were collected for each experiment and one control. H3K27me3 presents unique challenges for ChIP-seq analysis. Unlike transcription factors and certain other histone modifications, H3K27me3 forms broad patterns of enrichment that are of indeterminate length (genome-wide analysis has shown these to be up to ∼100 kb in *Drosophila*) (Papp and Müller 2006; Kharchenko et al. 2011; Nègre et al. 2011), and therefore simple peak-based analysis is not a suitable analysis method (Xiao et al. 2012). Because of the many distinct cell types of a *Drosophila* puparium, it is also not appropriate to treat H3K27me3 as a binary modification of chromatin (Zang et al. 2009). Therefore we chose to analyze the data quantitatively, using the number of sequence reads falling within prespecified intervals (e.g., the exons of a gene) as an indication of the overall level of H3K27me3 signal within a region (Supplemental Fig. S1). This measure is both highly correlated between biological replicates (Supplemental Fig. S2) and robustly anticorrelated with gene expression (Supplemental Fig. S3), suggesting that the metric is biologically meaningful. Using the modENCODE-curated list of orthologous genes in *Drosophila* (AP Boyle, CL Araya, C Brdlik, P Cayting, C Cheng, Y Cheng, K Gardner, L Hillier, J Janette, L Jiang, et al., in prep.), we directly compared each gene in the *melanogaster* genome to its ortholog in *D. simulans*, *D. yakuba*, and *D. pseudoobscura* (abbreviated Sim, Yak, and Pse). In total, we compared 12,017 orthologs from *D. melanogaster* to *D. simulans*; 11,018 from *D. melanogaster* to *D. yakuba*; and 11,881 from *D. melanogaster* to *D. pseudoobscura*.

**Figure 1.**
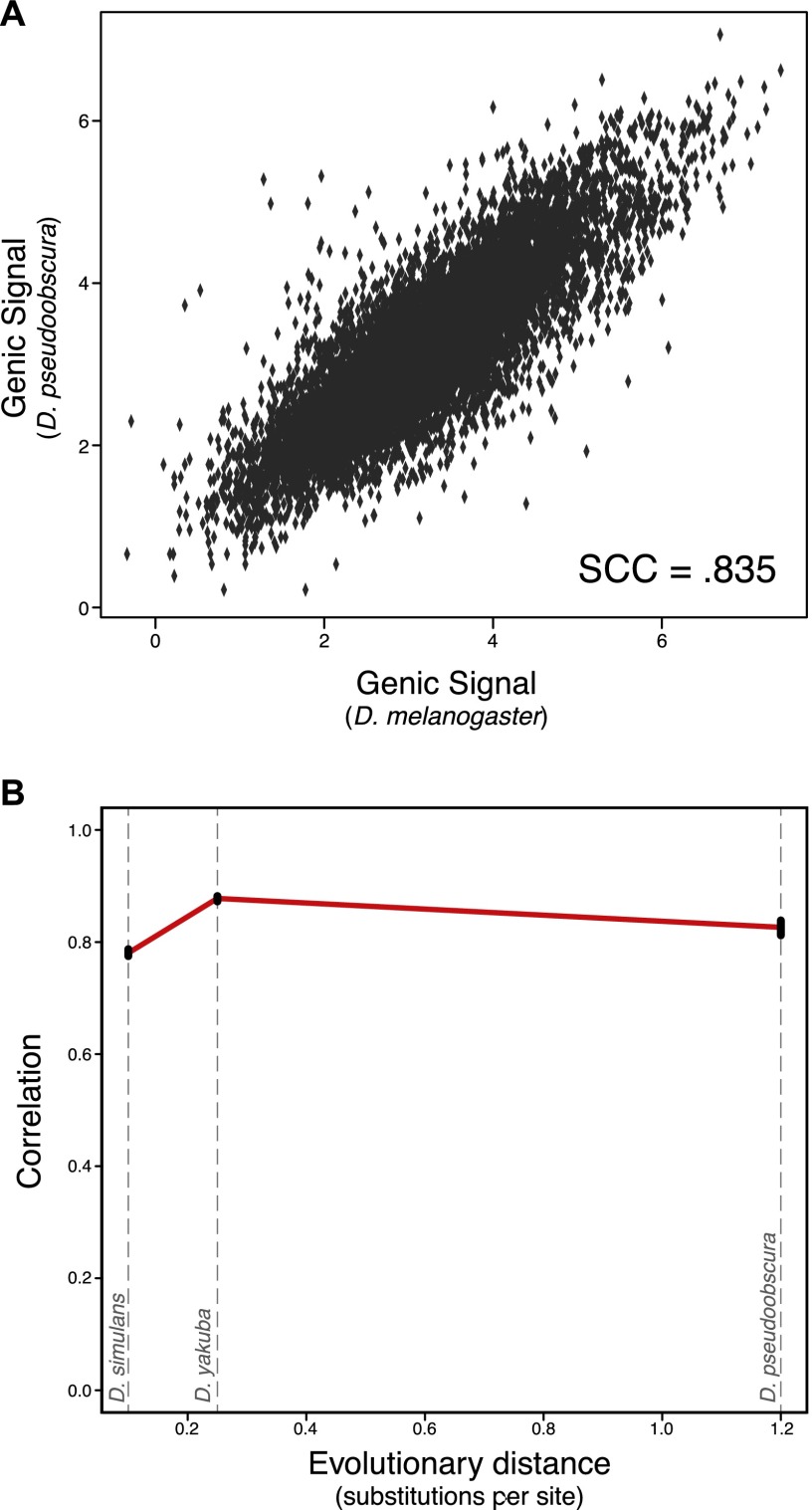
Strong conservation of H3K27me3 in *Drosophila* orthologs. (*A*) Example graph of orthologous gene conservation for the comparison of *melanogaster* to *pseudoobscura*. Each dot is a single-copy orthologous gene pair, and the position on the *x*-axis represents the log *melanogaster* genic signal (see Methods), while the *y*-axis represents the log *pseudoobscura* genic signal; each is the mean of two experiments. The overall rank correlation coefficient between species is 0.835. (*B*) Overall trend of single-copy ortholog conservation within *Drosophila*. Each point is the Spearman rank correlation of one pairwise between-species comparison, plotted against the evolutionary distance from *Drosophila melanogaster* (in substitutions per neutral site). Black bars represent bootstrapped 95% confidence intervals. From *left* to *right* the species are *D. simulans* (SCC = 0.781), *D. yakuba* (SCC = 0.878), and *D. pseudoobscura* (SCC = 0.835).

Overall we found high conservation of H3K27me3 signal between *Drosophila* genomes, ranging from Spearman correlation coefficients (SCC) of 0.78 to 0.88 (Fig. 1A; a representative locus is depicted in Supplemental Fig. S4). Given that any two pairs of biological replicate experiments show a SCC of ∼0.95 (Supplemental Fig. S2), the observed between-species correlations indicate relatively slow evolutionary change for H3K27me3 patterns across the genome. Simulations showed that a relatively small level of ortholog misidentification or misannotation error (10%–20%; similar to published estimates) (Chen et al. 2007; Creevey et al. 2011) is sufficient to explain the observed decrease in correlation between species relative to the technical variation within species. Notably, we do not see evidence for a linearly decreasing trend of conservation over phylogenetic distance within the three examined pairwise comparisons (Fig. 1B), in contrast to what has been observed for transcription factor binding sites in similarly low numbers of species (Dowell 2010; He et al. 2011; Ni et al. 2012).

Fold change in H3K27me3 of single-copy orthologs showed a weak correlation with accelerated sequence evolution. For example, change in H3K27me3 is positively associated with elevated *d*_N_/*d*_S_ (SCC = 0.045, permutation test, *P* < 10^−5^) in a comparison of *D. melanogaster* and *D. simulans*. These results are consistent with observations of mammalian stem cells (Xiao et al. 2012). Surprisingly, there is no strong correlation between change in H3K27me3 signal and change in gene expression among single-copy orthologous genes (SCC: Sim: 0.06; Yak: 0.043; Pse: −0.027). As noted above, this observation is consistent with most differences between single-copy orthologs being a result of technical variation. Alternatively, differences in H3K27me3 signal might be compensated for by other mechanisms of transcriptional regulation, resulting in little ultimate difference in gene expression.

### Conservation of H3K27me3 between *Drosophila* and *Caenorhabditis* orthologs

Given the extremely high conservation of the H3K27me3 epigenetic mark between *Drosophila* orthologs, we investigated whether H3K27me3 signal is conserved at greater phylogenetic distances. We chose to examine pairwise conservation between *D. melanogaster* and the nematode worm *Caenorhabditis elegans* (abbreviated Cel), whose most recent common ancestor dates to the Cambrian divergence (>500 Myr) (Nei et al. 2001). We compared our *D. melanogaster* prepupal data with corresponding H3K27me3 data generated by the modENCODE Consortium at the L3 stage in worms (results were similar when comparing embryonic stages as well; Supplemental Fig. S5). There were a total of 3157 single-copy orthologs between these two species.

**Figure 2.**
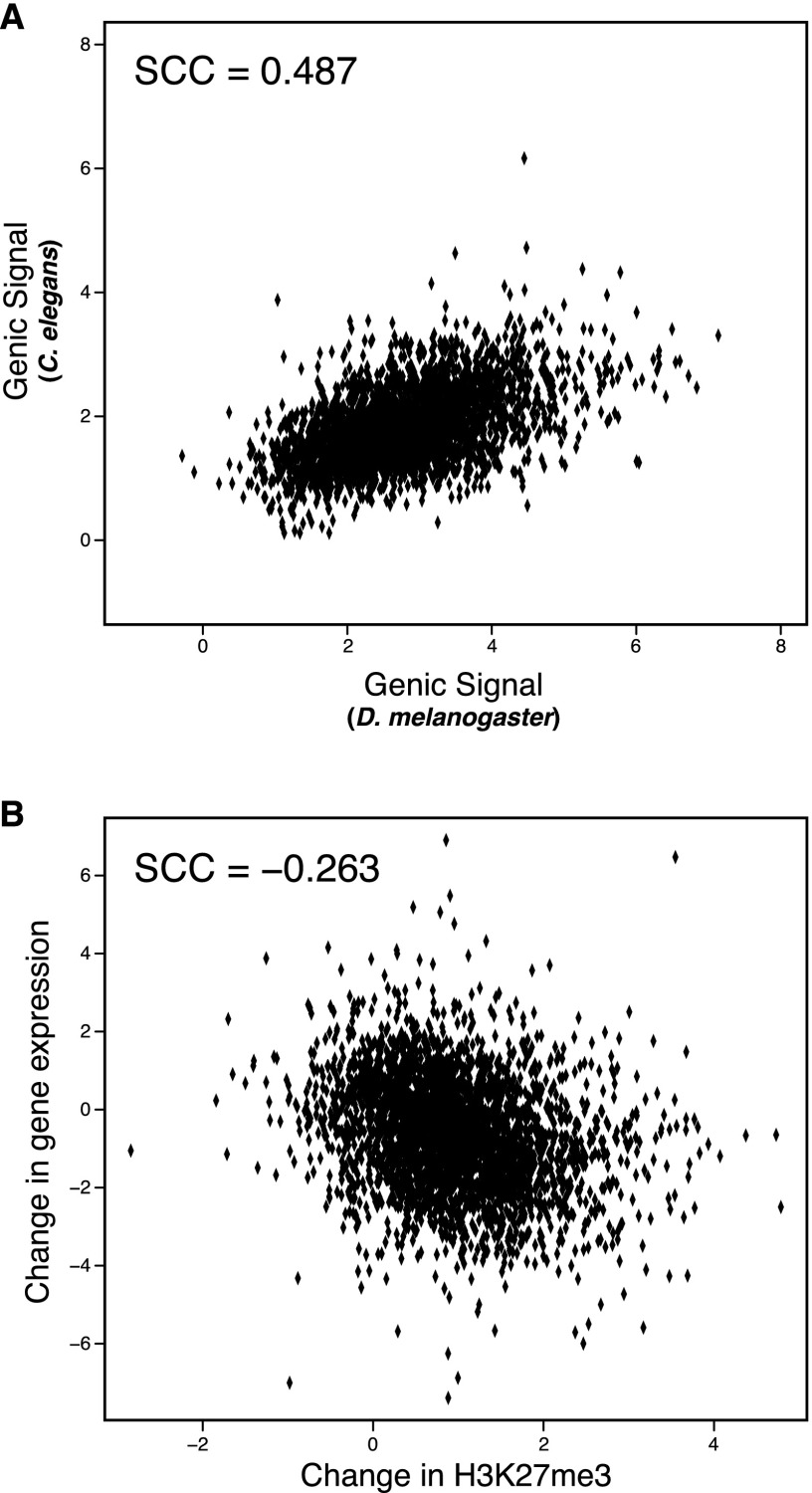
Conservation of H3K27me3 extends to *Caenorhabditis elegans*. (*A*) Substantial conservation of H3K27me3 between *D. melanogaster* and *C. elegans*. As in Figure 1A, each dot represents a gene, with the *x*-axis position corresponding to log H3K27me3 signal in *D. melanogaster* and the *y*-axis position corresponding to log H3K27me3 signal in *C. elegans*. The Spearman correlation coefficient is 0.487 between them. (*B*) Change in H3K27me3 levels is associated with changes in gene expression. Each dot is an individual single-copy orthologous gene, where the *x*-axis is the change in log H3K27me3 and the *y*-axis is the change in log expression. The overall Spearman correlation coefficient is −0.263.

We found substantial evidence of H3K27me3 conservation between these two highly diverged species (Fig. 2A) (SCC = 0.487); H3K27me3 is nearly as conserved as gene expression for these genes (SCC = 0.497). Assuming the lowest observed rate of loss in correlation observed within *Drosophila* and a linear relationship between divergence time and loss of correlation, one would expect no significant correlation given the phylogenetic distance separating worm and fly. H3K27me3 has previously been compared between cell lines from human, mouse, and pig, three species separated by 50–100 million years (Xiao et al. 2012). To our knowledge this is the first study to show significant conservation of an epigenetic mark between species as diverged as *D. melanogaster* and *C. elegans*. There is a moderate, significant negative correlation between gain of H3K27me3 and loss of expression (Fig. 2B) (SCC = −0.263). This result stands in contrast to the relative lack of correlation between expression change and H3K27me3 change within *Drosophila*, and may be a consequence of greater fold change when comparing *D. melanogaster*–*C. elegans* (median, absolute log_2_ fold change: 0.699) than *D. melanogaster* with, for example, *D. pseudoobscura* (median, absolute log_2_ fold change: 0.469).

### Evolution of H3K27me3 is more rapid in gene duplicates

Next we sought to examine how H3K27me3 signal might vary in the aftermath of gene duplication events. Gene duplication has been found to be a significant driver of regulatory and gene expression divergence in other interspecific comparisons (Gu et al. 2004; Kaessmann et al. 2009). While we found above that H3K27me3 is highly conserved among single-copy orthologs, we hypothesized that gene duplication might allow the creation of novel H3K27me3 regulation among duplicated gene orthologs. We therefore compared H3K27me3 levels for each newly duplicated gene to its corresponding single-copy ortholog in another genome (see Supplemental Fig. S6).

Because duplicated genes tend to share substantial nucleotide similarity, it is possible that some ChIP-seq reads might have difficulty mapping accurately to paralogs, either by mapping to the wrong paralog or failing to map uniquely and thus being discarded. To investigate the extent of this issue, we performed read simulation studies (Huang et al. 2012) which showed that most paralogs are accurately mappable, and we excluded from further analysis those genes that are not mappable (see Supplemental Methods; Supplemental Fig. S7). As a further precaution, we also performed read mapping in which up to two valid alignments are accepted for the pairwise comparison of *D. melanogaster* to *D. yakuba*; all results remained significant.

To call individual genes, either duplicated or single-copy orthologs, as significantly diverged in H3K27me3 signal (relative to the orthologous copy in the other genome), we quantified the degree of H3K27me3 divergence and compared to a permutation-based null (see Supplemental Methods). Significantly evolved genes (FDR of 0.05) showed greater absolute differences in expression between species (Permutation test: Sim: *P* = 0.11; Yak: *P* < 10^−5^; Pse: *P* < 10^−5^), and higher *d*_N_/*d*_S_ (Permutation test: Sim: *P* < 10^−5^).

**Figure 3.**
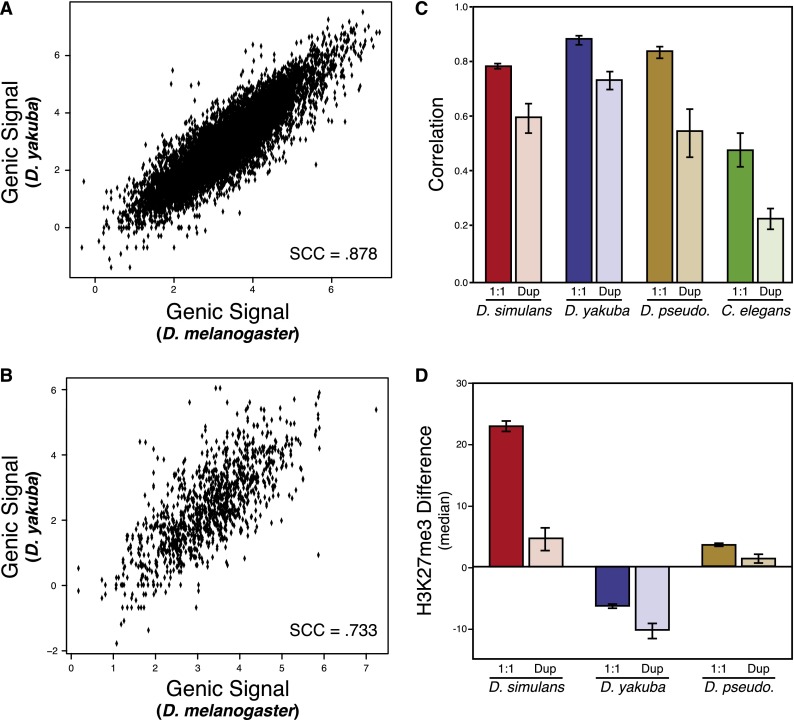
Duplicated genes have less conserved H3K27me3 signal. (*A*) As in Figure 1A, single-copy orthologous gene H3K27me3 conservation between *melanogaster* and *yakuba*. (*B*) As in *A* and Figure 1A, but depicting duplicated gene orthologs. (*C*) Overall pattern of H3K27me3 conservation in different gene sets. Each pair of bars represents one species comparison; bars on the *left* are the correlation coefficient of single-copy orthologs; on the *right*, the correlation coefficient of duplicated gene orthologs. Each bar represents a single pairwise comparison’s Spearman rho, compared against *Drosophila melanogaster*: (red) *simulans*; (purple) *yakuba*; (brown) *pseudoobscura*; (green) *C. elegans*. Overall correlation decreases in each case. Lines are 95% bootstrapped confidence intervals for each correlation; we account for the differences in sample size (single-copy orthologs are more common than duplicated gene orthologs) by resampling the number of duplicated gene orthologs in each case. (*D*) Duplicated gene orthologs are more likely to lose H3K27me3. Each pair of barplots is one species (*simulans*, *yakuba*, *pseudoobscura*); the *left* in each pair is the median H3K27me3 change in duplicated genes relative to the single gene in the other genome; while the *right* is the median in single-copy orthologs. Each pair shows that duplicated genes are biased toward the loss of H3K27me3 signal. For each pair (by permutation test): *simulans*, *P* < 10^−5^; *yakuba*, *P* < 10^−5^; and *pseudoobscura*, *P* < 10^−5^.

H3K27me3 conservation differs systematically depending upon the occurrence of gene duplications. Duplicated gene orthologs generally have lower conservation of H3K27me3 signal, relative to the single-copy orthologs (Fig. 3A–C) (this is true even after accounting for differences in number of duplicated vs. single-copy orthologs). In each species comparison, duplicated genes constitute three to four times more of the H3K27me3-diverged set than expected based on their overall frequency in the compared genomes (Table 1) (Fisher’s exact test: Sim: *P* = 3 × 10^−12^; Yak: *P* < 2.2 × 10^−16^; Pse: *P* = 6.9 × 10^−11^). Based on these results, we infer that H3K27me3 signal is more labile following gene duplication events.

**Table 1.**
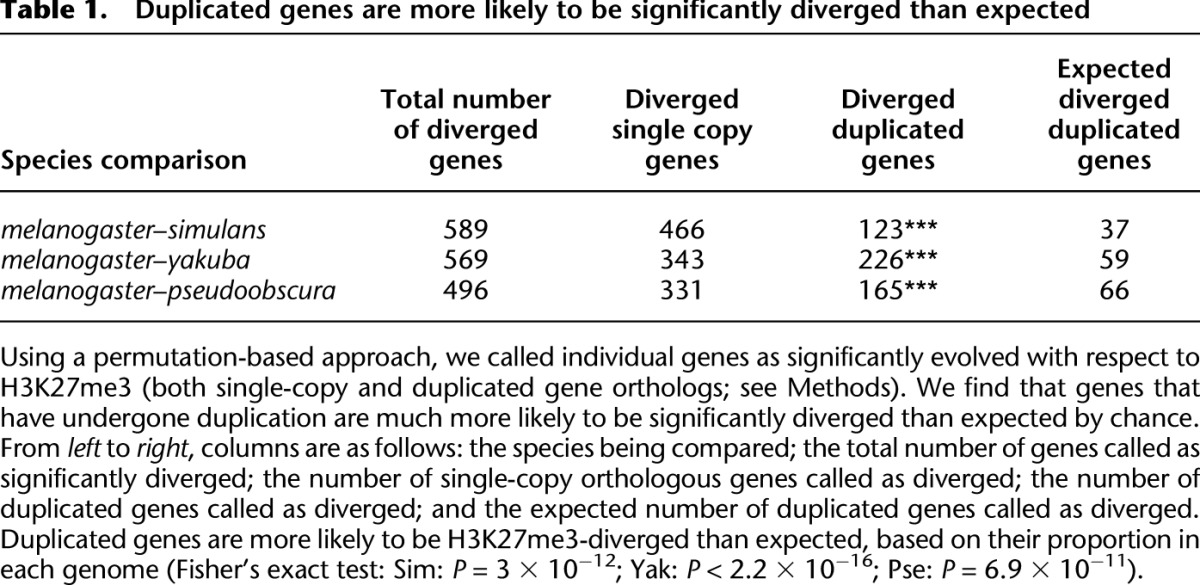
Duplicated genes are more likely to be significantly diverged than expected

While duplicated gene orthologs tended to have less confidence in orthology assignment, we did not find a significant association between ortholog bootstrap confidence and magnitude of change in H3K27me3, indicating that poor ortholog identification is not responsible for the observed trend (Supplemental Fig. S8). In addition, excluding large ortholog families that had experienced many duplications did not change the trend significantly (Supplemental Fig. S9). To further verify that read mismapping within paralogs was not the cause of our results, we analyzed the promoters of each orthologous gene similarly. Because promoter sequences evolve more rapidly than exonic sequence, promoters should suffer less from read mismapping. We found that, as with exonic H3K27me3 signal, promoters’ methylation signal conservation was less for duplicated gene orthologs than for single-copy orthologs (Supplemental Fig. S10).

While change in H3K27me3 was associated with accelerated sequence evolution in single-copy orthologs, this effect was much more pronounced in duplicated gene orthologs (SCC = 0.099 for duplicated genes vs. SCC = 0.045 for single-copy orthologs; this difference is significant [by bootstrap, *P* < 0.05]). Similarly, duplicated gene orthologs showed stronger correlations between gain of H3K27me3 and loss of expression (Permutation test: Sim, *P* < 10^−4^; Yak, *P* < 10^−4^; Pse, *P* < 10^−4^). The more robust associations of H3K27me3 evolution, gene expression evolution, and *d*_N_/*d*_S_ in duplicated gene orthologs may indicate a tighter relationship between H3K27me3 evolution and neofunctionalization in duplicated genes than in single-copy orthologs.

Comparing duplicated genes to each other (rather than orthologous genes in another genome), we found that duplicated gene paralogs are tightly correlated in the direction and magnitude of H3K27me3 evolution after a duplication event. Evolution of H3K27me3 in duplicated gene paralogs showed significant positive correlations, indicating that both duplicates tended to undergo similar epigenetic changes after their duplication (Sim: 0.264, Yak: 0.362, Pse: 0.348, Cel: 0.447). The positive correlation indicates that the duplication event allows not only the new gene to alter its epigenetic state, but also the parent.

The median H3K27me3 change for duplicated orthologs is significantly lower than single-copy orthologous gene H3K27me3 differences in all cases (Fig. 3D), indicating an overall loss of H3K27me3 following duplication (Permutation test: Sim, *P* < 10^−5^; Yak, *P* < 10^−5^; Pse, *P* < 10^−5^). However, we observed that there were significant positive correlations between gene duplicate age and evolutionary change of H3K27me3 levels, indicating that gene duplicates regain lost H3K27me3 signal as they age (SCC: Yak: 0.109, *P* < 10^−5^; Pse: 0.064, *P* = 0.015). The loss of H3K27me3 was also related to an average increase in expression of both duplicates (Permutation test: Sim, *P* < 10^−5^; Yak, *P* < 10^−5^; Pse, *P* < 10^−5^). We examined expression patterns in newly duplicated genes using the Berkeley *Drosophila* Genome Project’s in situ database (Tomancak et al. 2002) and found that such genes are enriched for tissue-specific expression (Fisher’s exact test, *P* = 0.0006). Since these recent duplicates generally lose H3K27me3, our results imply that such genes may acquire tissue specificity through DNA-binding *trans*-regulators.

**Figure 4.**
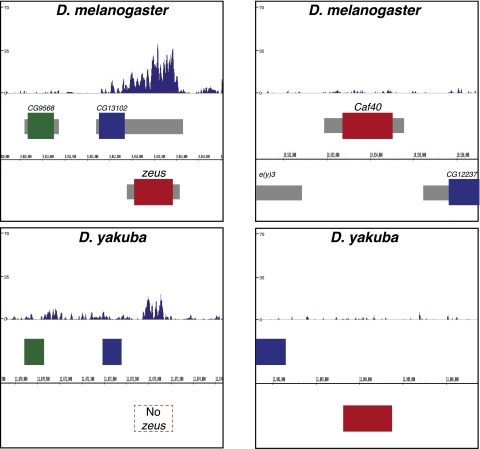
The novel gene duplicate *Zeus* has undergone rapid gain of H3K27me3. (*Left*) A Genome Browser snapshot indicating H3K27me3 signal at the *Zeus* (*Rcd-1r*) locus in *D. melanogaster*. The first window indicates the normalized, input-subtracted H3K27me3 signal pooled from two separate biological replicates in *D. melanogaster*. (*Below*) Boxes indicate protein-coding genes: CG9568 (green), CG13102 (blue), and *Zeus* (red). The next window is as above, but in an outgroup species (*D. yakuba*), which does not possess the *Zeus* retroposition. Browser snapshots are aligned such that orthologous genes match position. Note that the H3K27me3 level around *Zeus* is substantially higher in *D. melanogaster* relative to the equivalent region in *D. yakuba*. (*Right*) As above, but focusing instead on the parental gene *CAF40* (*Rcd-1*; red), flanked on the *left* by *e(y)3* (gray), and on the *right* by CG12237 (blue). Note that *CAF40* possesses little to no H3K27me3 signal, in strong contrast to *Zeus*. The sequence identity between *Zeus* and *CAF40* is 71% (Quezada-Díaz et al. 2010).

A notable exception to this pattern is the gene *Zeus* (FBgn0032089; also called *Rcd-1r*). Previously, *Zeus* has been shown to be a recent duplicate of *CAF40* (FBgn0031047; also called *Rcd-1*), which has acquired an essential role in gonadal development (Chen et al. 2012). *Zeus* duplicated sometime before the common ancestor of *D. melanogaster* and *D. simulans*; it is not present in *D. yakuba*. *Zeus* has acquired a substantial increase in H3K27me3 in *D. melanogaster* relative to both its paralog, *CAF40*, and the location into which it duplicated in the *D. yakuba* genome (Fig. 4).

### Duplicated genes relocate to regions of low H3K27me3 signal

Novel genes, once duplicated, must rapidly acquire unique functionality or otherwise suffer pseudogenization (Force et al. 1999). However, to acquire functionality a gene must first be expressed and exposed to selection. We predicted that newly duplicated genes in repressive chromatin environments (bearing high H3K27me3 signal) would be less expressed and thus more likely to become pseudogenes.

**Figure 5.**
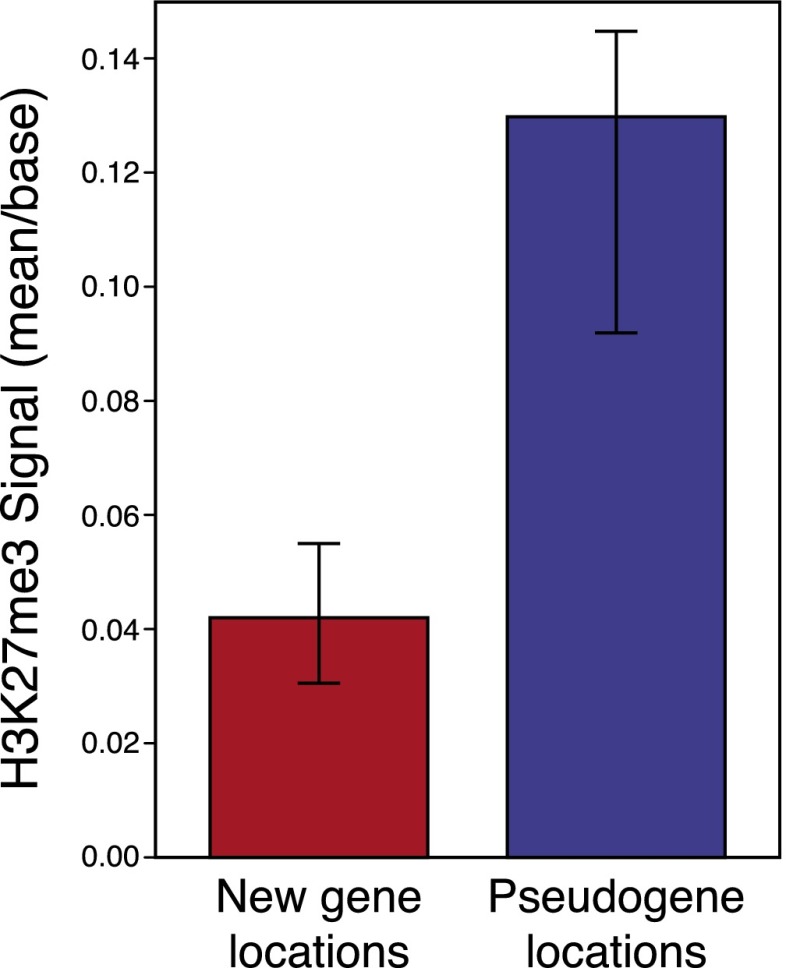
Locations into which duplicates move affects eventual pseudogenization fate. Regions in the *D. simulans* genome into which new genes and pseudogenes had moved in *D. melanogaster* show differences in mean H3K27me3 signal. On the *left*, functional new genes’ duplication locations show significantly less H3K27me3 signal than pseudogenes’ equivalent locations. Bars represent bootstrapped 95% confidence intervals. Note that for the purposes of this analysis, we assume that extant *D. simulans* H3K27me3 signal is representative of the H3K27me3 signal occurring in the ancestor of *D. melanogaster* in which new gene duplications occurred (see Methods).

We examined the relative H3K27me3 profiles in *D. simulans* for the locations into which both *D. melanogaster*-specific novel genes and pseudogenes had duplicated. Using synteny, we located the regions in *D. simulans* that novel genes moved into to infer the ancestral H3K27me3 levels before the duplication occurred (see Supplemental Methods). Importantly, this analysis relies on the assumption that the extant *D. simulans* H3K27me3 signal is, on average, similar to the H3K27me3 signal in the ancestor of *D. melanogaster* in which the gene duplication event occurred. We found that locations into which pseudogenes had duplicated were characterized by threefold more H3K27me3 signal than the locations into which novel, protein-coding genes had duplicated (Permutation test, *P* = 0.0015) (Fig. 5). This finding is consistent with our hypothesis, and suggests that chromatin-mediated silencing of novel gene expression can prevent the acquisition of function in newly duplicated genes, adding a new dimension to the duplication–degeneration–complementation model (Force et al. 1999). Furthermore, this result provides a mechanistic explanation for the observed bias toward loss of H3K27me3 in recent duplicated genes: Only those duplicates which move into regions of low H3K27me3 signal are likely to remain functional.

### Patterns of H3K27me3 evolution depend on duplication mechanism

Gene duplication events are known to occur via diverse mechanisms (Hastings et al. 2009), which lead to different consequences for the resulting duplicated gene’s location. Because H3K27me3 is spatially localized (Kharchenko 2011; Nègre et al. 2011), we might expect that localized gene duplication events (e.g., tandem duplication) would lead to the newly duplicated gene falling within or close to the parent gene’s H3K27me3 domain. Alternatively, a mechanism such as retroposition, which is able to deposit novel genes as far from their parent as different chromosomes (Kaessmann et al. 2009), might be able to completely change a novel gene’s epigenetic regulation by delivering the novel gene to a location with an entirely different chromatin profile.

**Figure 6.**
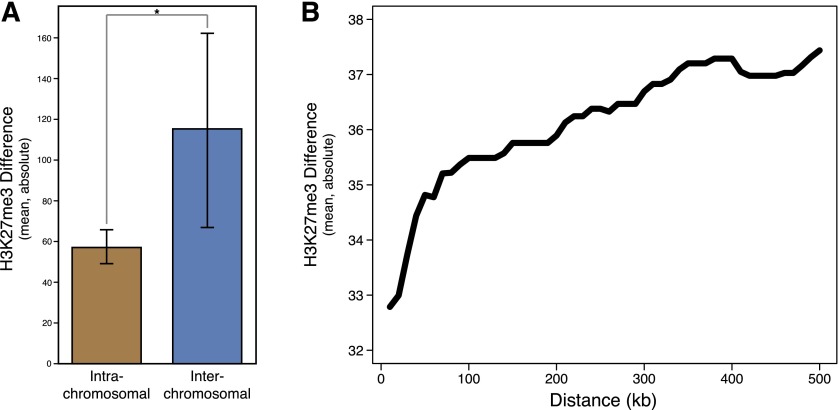
H3K27me3 changes in paralogs differ depending on duplicate location. (*A*) Within *Drosophila simulans*, interchromosomal translocations have significantly greater H3K27me3 changes (in terms of absolute value; permutation test, *P* < 10^−5^). To accurately identify chromosomal translocations, we limited comparison to the major chromosome arms (2R, 2L, 3R, 3L, and X), and considered any gene that moved between chromosome arms an interchromosomal translocation. (*B*) On the same chromosome, duplicated gene orthologs’ H3K27me3 changes increase with distance from the parent gene. On the *y*-axis, the cumulative mean change in H3K27me3 signal as a function of distance (on the *x*-axis). There is a significant correlation between distance and magnitude of H3K27me3 change (SCC = 0.103, *P* = 0.002).

We examined whether the distribution of H3K27me3 changes varies by the location of the duplicated gene relative to its parent. We found that interchromosomal translocations show greater H3K27me3 divergence than intrachromosomal translocations (Fig. 6A) (Sim, *P* < 10^−5^; Yak, *P* < 10^−5^). As expected, interchromosomal translocations also show greater divergence in gene expression (Sim, *P* = 0.017; Yak, *P* < 10^−5^). These results show that interchromosomal duplications drive greater change in H3K27me3 signal and concomitant divergence in gene expression.

The hypothesis that localization affects H3K27me3 divergence also predicts that intrachromosomal duplicates are more likely to undergo more extensive H3K27me3 evolution the further they are from the parent gene. Indeed, we see evidence within the intrachromosomal population that the distance between the old gene and the new gene is related to the subsequent H3K27me3 divergence (Fig. 6B). There is a significant correlation between distance from the parental gene and the magnitude of H3K27me3 divergence (Yak: SCC = 0.094; Permutation test, *P* = 0.001), but it is worth noting that this effect saturates after ∼100 kb, suggesting that moving beyond that threshold does little to further change H3K27me3 regulation. This saturation effect coincides with a loss of H3K27me3 autocorrelation in approximately the same range (data not shown).

## Discussion

Previous studies have discovered relatively slow evolution of histone modifications (Cain et al. 2011). Our results reinforce the slow evolution of a histone modification, but we find very different evolutionary regimes between single-copy orthologs and orthologs that have undergone duplication events. While single-copy orthologs exhibit relatively little divergence in terms of genic H3K27me3, duplicated genes evolve comparatively rapidly.

These differences are exaggerated in the most distant comparison we make, between *Drosophila melanogaster* and *Caenorhabditis elegans*. Orthologous genes in these species show substantial similarity in H3K27me3 signal, even though the two species are deeply diverged. Meanwhile, orthologous gene sets in which duplication events have occurred are significantly less conserved.

Among duplicated genes, we find interesting dynamics of H3K27me3 change. Gene duplicates show highly correlated gain or loss of H3K27me3 signal, indicating that both parent and duplicate undergo similar changes to their epigenetic status following duplication. Duplicated genes are biased with respect to the H3K27me3 change they are subject to: Both duplicates are more likely to lose methylation than single-copy orthologous genes. By examining the regions into which functional new genes localize relative to pseudogenes, we show that new genes tend to move to regions of low H3K27me3 signal. This result comports with the duplication–degeneration–complementation theory (Force et al. 1999), and suggests the importance of chromatin state as a determinant of novel gene fate. We speculate that the removal of H3K27me3 could allow paralogs to acquire new tissue specificity via *trans*-regulatory factors instead of histone modifications (Huerta-Cepas et al. 2011).

We find evidence that gene duplications on different chromosomes and at greater distances are more likely to acquire H3K27me3 signal and gene expression changes. The novel gene *Zeus* exemplifies this pattern, and has gained a substantial level of H3K27me3 relative to its parent gene *CAF40*. These results strongly imply that gene duplication mechanisms that can move the resulting duplicate further from the original gene are associated with more H3K27me3 evolution in the duplicate, which could have important consequences for understanding the eventual fate of gene duplicates. We do not know to what extent these differences would manifest for other histone marks, which tend to be relatively more compact than H3K27me3 domains (Kharchenko et al. 2011; Nègre et al. 2011).

Significant evolution of H3K27me3 signal was associated with evolution of gene expression and sequence evolution (as measured by *d*_N_/*d*_S_). In a separate comparison, independent of genes, a bin-based approach also found that evolution of H3K27me3 was also associated with an elevated rate of sequence substitution between species (see Supplemental Material; Supplemental Fig. S11). It appears that evolution of this mark is associated with evolution in the underlying sequence, although the causal basis of that association is ambiguous in the present study (Xiao et al. 2012). Whether H3K27me3 evolution allows the sequence to evolve, or whether H3K27me3 divergence is the result of new Polycomb regulation due to the creation or destruction of PcG binding sites is unknown.

To this end, future work is necessary to clarify exactly how the evolution of H3K27me3 is mediated genetically. Although many of the key enzymes responsible for deposition and maintenance of H3K27me3 are known, it is likely that we do not have a complete catalog of all the elements of all PREs (Delest et al. 2012), nor do we know the exact spatial or orientation requirements of the involved binding sites (if indeed there are any). However, the extreme conservation of H3K27me3 across distantly related species leads to the question of how a *cis*-regulatory element composed of relatively degenerate sequence motifs could persist functionally over such a long period of time (Ruvinsky and Ruvkun 2003; Fisher et al. 2006).

It is worth noting caveats that apply to the above analysis. Our results are gathered at only one strictly defined time period, which was chosen for its ease of use and developmental significance. Genes for which we observe no change in H3K27me3 signal may instead be highly diverged at other developmental times. Furthermore, it is possible that the dynamics of histone modification evolution differ depending on the stage of development (Kalinka et al. 2010). Additionally, we believe the observed differences in H3K27me3 reflect a lower bound estimate of the possible differences between species. Because of the heterogeneous nature of the sample material, it may be difficult to detect small-scale, tissue-specific differences in the epigenetic markings over loci. It may well be that orthologous genes undergo many changes of this nature between the species examined.

In summary, we have shown that H3K27me3 signal diverges more rapidly in duplicated genes. When diverged, duplicated genes most often show correlated loss of H3K27me3 relative to the single ortholog in the other genome, but the extent to which this mark changes depends on how far the duplicates are moved from the parent gene.

These results are indicative of an interesting dichotomy in the regulation of epigenetic states. Whereas single gene orthologs remain locked into their epigenetic status, duplicates are able to undergo rewiring of H3K27me3 signal and resulting gene expression. Our results indicate an interplay between gene duplication and the evolution of chromatin state as a mechanism for generating evolutionary novelty.

## Methods

### ChIP-seq

Strains were maintained at room temperature until collection at the white prepupa stage. ChIP-seq was carried out in duplicate using the standard modENCODE protocols. We mapped 36-bp reads to the appropriate reference genome using Bowtie (v.0.12.7) (Langmead et al. 2009). *Caenorhabditis elegans* H3K27me3 ChIP-seq data were generated as described in AP Boyle, CL Araya, C Brdlik, P Cayting, C Cheng, Y Cheng, K Gardner, L Hillier, J Janette, L Jiang, et al. (in prep.). Data from Nègre et al. (2011) are available under accession numbers GSE27111 and GSE23537.

### RNA-seq

Single-end reads were trimmed and mapped using TopHat, and FPKM values were called with Cufflinks (Trapnell et al. 2012). *Caenorhabditis elegans* RNA-seq data were processed as described in Li et al. (2014).

### Quantitative analysis

We used BEDTools (Quinlan and Hall 2010) to count the number of reads occurring in either (1) the exons of each gene as determined by the reference annotation, or (2) regular sliding windows 1 kb in width (Quinlan and Hall 2010). Genic H3K27me3 was normalized by the number of exons (see Supplemental Methods). We compared orthologous genes using the list of modENCODE orthologs, and we compared orthologous windows by using liftOver to map windows between species.

### Pseudogene location analysis

In order to examine the H3K27me3 signal occurring in the locations of recently duplicated genes/pseudogenes, we extracted the two flanking regions (both 500 bp) around each recently duplicated gene or pseudogene, which were then lifted into the *D. simulans* genome using liftOver. To limit our analysis to very recent, confident duplication events, we only analyzed the comparison of *D. melanogaster*–*D. simulans*, and considered only cases in which both flanking regions mapped, with a distance of <5 kb between them. We examined H3K27me3 levels in the region between flanks as representative of the ancestral state (see Supplemental Methods).

### Statistical analysis

To call individual intervals as significantly diverged in different species, we used DESeq (Anders and Huber 2010) on the control-subtracted read counts within bins. To estimate a false discovery rate (FDR), we used the *Q*-value package in R (Storey and Tibshirani 2003). To estimate the significance of differences between two populations’ sample means, we used permutation tests as described in Sokal and Rohlf (1994) (p. 808; called therein “sampled randomization” tests). All statistical analysis was performed in R.

## Data access

*Drosophila* RNA-seq data and ChIP-seq data from this study have been submitted to the NCBI Gene Expression Omnibus (GEO; http://www.ncbi.nlm.nih.gov/geo/) under accession number GSE49945. *Caenorhabditis* ChIP-seq data have been submitted to GEO under accession numbers GSE49724 and GSE49738.
